# Valosin-Containing Protein, a Calcium-Associated ATPase Protein, in Endoplasmic Reticulum and Mitochondrial Function and Its Implications for Diseases

**DOI:** 10.3390/ijms21113842

**Published:** 2020-05-28

**Authors:** Xiaonan Sun, Hongyu Qiu

**Affiliations:** Center of Molecular and Translational Medicine, Institution of Biomedical Science, Georgia State University, Atlanta, GA 30303, USA; xsun13@gsu.edu

**Keywords:** endoplasmic reticulum, mitochondria, valosin-containing protein, calcium homeostasis, disease

## Abstract

Endoplasmic reticulum (ER) and mitochondrion are the key organelles in mammal cells and play crucial roles in a variety of biological functions in both physiological and pathological conditions. Valosin-containing protein (VCP), a newly identified calcium-associated ATPase protein, has been found to be involved in both ER and mitochondrial function. Impairment of VCP, caused by structural mutations or alterations of expressions, contributes to the development of various diseases, through an integrating effect on ER, mitochondria and the ubiquitin–proteasome system, by interfering with protein degradation, subcellular translocation and calcium homeostasis. Thus, understanding the role and the molecular mechanisms of VCP in these organelles brings new insights to the pathogenesis of the associated diseases, and leads to the discovery of new therapeutic strategies. In this review, we summarized the progress of studies on VCP, in terms of its regulation of ER and mitochondrial function and its implications for the associated diseases, focusing on the cancers, heart disease, and neurodegenerative disorders.

## 1. Introduction

The endoplasmic reticulum (ER) is one of the largest membrane organelles in cells, and plays an important role in protein synthesis, protein folding and quality control, lipid metabolism and Ca^2+^ homeostasis [1]. As ER is found in all cell types, the sarcoplasmic reticulum (SR), a morphologically distinct version of the ER, only exits in muscle cells that are specialized for Ca^2+^ release to fuel muscle contraction. SR consists of two spatial and functional organizations, termed longitudinal SR and junctional SR, tasked with the release and the uptake of Ca^2+^, ensuring delivery of Ca^2+^ for contraction to occur [2,3,4]. ER dysfunction has been associated with cellular dysfunction and cell death [5]. ER contains multiple domains, among which the biggest is a bilayer barrier around the cell nucleus, named the nuclear envelope. In addition, the peripheral ER membrane extends from the nuclear envelope to the whole cytoplasm, forming interactions with other organelles and contacting the plasma membrane [6]. One of the most essential organelles that the ER interacts with is the mitochondria, which are responsible for regulating Ca^2+^ homeostasis, apoptosis and ATP production. The dysfunction of mitochondria leads to impaired energy production and contributes to the pathogenesis of many metabolic diseases [7,8]. Most recent evidence has demonstrated that mitochondria communicate with the ER via mitochondria-associated ER membranes (MAMs) [8,9]. Although increasing evidence demonstrates that impaired function of the ER and mitochondria is associated with the pathogenesis of a variety of diseases, including cancers, neurodegeneration diseases and heart disease, as well as metabolic diseases such as obesity and diabetes [9,10,11], the underlying mechanisms remain largely unknown.

The valosin-containing protein (VCP), also known as p97 in mammals, Cdc48 in yeast and plants, CDC-48 in worms and Ter94 in flies, was initially recognized as one of the ER-associated proteins, and has been demonstrated as playing critical roles in regulating ER formation and morphology by participating in the ubiquitin–proteasome system (UPS) and other intracellular signaling pathways [12,13]. One of the key functions of VCP is to conjugate its substrates with the ubiquitin chain through interaction with a variety of ubiquitin adapters, and then transport the substrates to the 26S proteasome for subsequent degradation, such as ER-associated protein degradation (ERAD) [13]. The mutations of VCP are found to be associated with some human degenerative disorders, such as amyotrophic lateral sclerosis (ALS), inclusion body myopathy (IBM) associated with Paget disease of the bone (PDB), and frontotemporal dementia (FTD), also called IBMPFD, which mainly affect the brain and muscles [12]. In addition, the VCP expression level was also shown to be upregulated in some cancers, mainly in response to the increased burden of protein degradation, indicating that VCP inhibition could be a promising therapeutic approach to cancer management [13,14,15]. Furthermore, recent studies also found that VCP participates in cardiomyocyte growth and survival, and plays a protective role against the stress-induced pathogenesis in heart diseases by attenuating mitochondrial and ER stress through regulating calcium homeostasis [16,17,18,19]. 

In this review, we summarized the new progress achieved in studies of VCP, regarding its regulating effect on ER and mitochondria functions, and its implications for various diseases, focusing on cancer, heart disease and neurodegenerative disorders.

## 2. The Structure and Distribution of VCP in Mammal Cells

**Figure 1:**
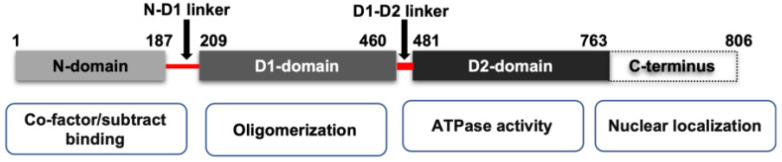
The scheme of the mammalian isoform of VCP domains and their function. VCP is constituted by one binding domain N-domain, two ATPase domains (D1 domain and D2 domain) and a C-terminus. N-domains are responsible for substrate recognition and binding. The D2 domain contributes to the major ATPase activity of VCP, while the D1 domain is responsible for the assembly of VCP homohexamer. The N-domain and D1 domain are connected by an N-D1 linker, and the D1 and D2 domains are connected by D1–D2 linker.

VCP belongs to the type II class of the AAA (ATPases Associated with various cellular Activities) ATPase family [20]. As shown in Figure 1, VCP possesses four structural domains, including a conserved N-terminal domain, two AAA ATPase domains (D1 and D2), and a C-terminal tail. D1 and D2 domains are stacked in a head-to-tail manner and connect with a short polypeptide linker, while the N-terminal domain is connected to the D1 domain by another short linker. In mammalian cells, VCP functions as a homohexamer [20]. The active form of VCP is a complex of a double ring structure, with the D1 and D2 domains sitting on top of each other. 

While the N-terminal domain of VCP is involved in the substrate’s recognition and interaction with other cofactors, the C-terminal tail was shown to be involved in nuclear localization via interacting with other proteins. Meanwhile, the D2 domain undertakes the major ATPase activity, while the D1 domain mainly contributes to the assembly of the hexamers [20]. The linkers between the D1 and D2 domains, and the D1 and N terminal domains, are also reported to be critical to the functions of VCP. For instance, the linker region between the D1 and D2 domains is essential for the D2 ATPase activity [21]. Interestingly, most disease-associated VCP mutations are found in the N-domains and ATPase domains [22], and those mutations in VCP have been shown to impair mitochondrial function and protein homeostasis [23,24]. It also reported that the mutations in the D1–N terminal linker region of VCP may cause neurodegenerative disorders in humans [25].

VCP is one of the most broadly expressed proteins in human, and it can be detected in a variety of organs such as the brain, skeletal muscle, heart, kidney, liver, ovary, testis and lung [26]. In mammalian cells, VCP is found to be distributed in different subcellular organelles, such as cytoplasm, Golgi apparatus [27], nuclear envelope [28], ER and mitochondria [29]. It has been shown that VCP, an ATPase, participates in a diverse array of cellular functions, including DNA replication, protein folding and degradation, the ubiquitin–proteasome system (UPS), calcium homeostasis, chromatin remodeling, and the assembly of Golgi and nuclear membranes [30]. 

## 3. The Regulation of VCP in ER and Mitochondrial Function in Physiological Condition

The function of ER under normal conditions is responsible for regulating protein folding and synthesis, post-translational modification, and maintaining the transaction of different transmembrane proteins. It has been shown that VCP, as a ubiquitin-selective chaperone, plays an essential role in maintaining ER integrity through interacting with E3 ubiquitin ligases, such as Glycoprotein 78 (gp78, also known as AMFR) and ERAD-associated E3 ubiquitin-protein ligase HRD 1 (Hrd1) [31,32]. 

Under normal conditions, in order to restore ER homeostasis, accumulated misfolded proteins in the ER active the signal of the unfolded protein response (UPR), which initiates the ERAD process through activating transcription factor 6 (ATF6), inositol-requiring enzyme 1α (IRE-1 α) and the PKR-like ER kinase (PERK) signaling pathways [33], by which the misfolded proteins are exported from ER to cytoplasm, where those proteins are subject to proteasomal degradation. Studies have shown that VCP plays a critical role in maintaining the homeostasis of ER, by regulating ubiquitin-dependent processes through the ubiquitin–proteasome system (UPS). By interacting with various polyubiquitinated proteins, such as ubiquitin ligase gp78, ubiquitin fusion degradation 1L, UBX-domain containing protein 1 (UBXD1), p47, nuclear protein localization protein 4 (Npl4), or other ubiquitin-like proteins, VCP is involved in the regulation of the activity of these factors and the delivery of misfolded proteins for proteasomal degradation [29,32,34]. VCP also interacts and binds to the ubiquitinated substrates, and regulates the downstream of ubiquitylation [35]. During ERAD, VCP extracts ubiquitinated misfolded proteins from ER membranes to the 26S proteasome for degradation, preventing the misfolded proteins’ aggregation, which is the main cause of ER stress.

The ER physically directly interacts with mitochondria, and plays a critical role in maintaining mitochondrial function. For instance, VCP can extract misfolded proteins from mitochondria during the process of mitochondria-associated degradation with the VCP cofactors Ufd1-Np14. VCP has been shown to participate in mitophagy-related degradation, which depends on the E3 ligase Parkin [36]. VCP is also required for the retro-translocation of the anti-apoptotic protein myeloid cell leukemia sequence 1(Mcl1) from mitochondria to cytosol for the proteasomal degradation [29]. 

In addition, ER–mitochondria tethering plays a critical role in regulating calcium homeostasis through MAM. The inositol triphosphate receptor (IP3R), a Ca^2+^ channel on ER, is highly accumulated in MAM [37]. Through the interaction of the regulators and the Ca^2+^ channel regulation protein complex, Ca^2+^ transfer can be modulated between ER and mitochondria [38,39]. In mitochondria, different Ca^2+^ levels trigger different molecular activities. Increase of the mitochondrial Ca^2+^ stimulates electron activity and ATP generation [40]. However, excessive Ca2+ levels will trigger apoptosis, through inducing mitochondrial permeability transition pore (mPTP) opening [41]. Thus, the mechanism of VCP in regulating mitochondrial calcium homeostasis has attracted attention. Recent studies have shown that VCP participates in mitochondrial respiration, calcium intake and mPTP, through regulating the mitochondrial calcium uptake (MICU) proteins by regulating the degradation of these proteins [17]. 

**Figure 2:**
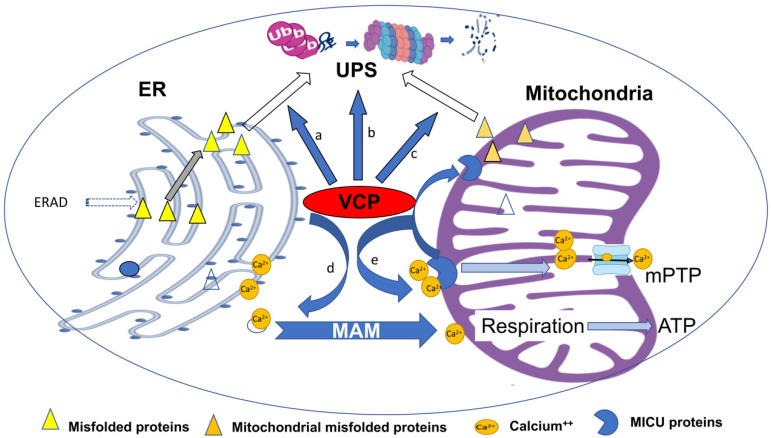
Summary of the integrating regulation of VCP on ER- and mitochondrial-associated degradation and calcium homeostasis. VCP plays an essential role in maintaining cellular function and calcium homeostasis in physiological conditions through multiple mechanisms. By interacting with other proteins, VCP participates in the delivery of misfolded proteins from the ER (a) and promotes the ubiquitin-dependent degradation processes through the ubiquitin–proteasome system (UPS) during ERAD (b). VCP is also involved in extracting the misfolded proteins from mitochondria during the process of mitochondria-associated degradation, and participates in mitophagy-related degradation (c). VCP participates in the regulation of calcium homeostasis through mitochondria-associated ER membranes (MAMs), which stimulates electron activity and ATP production (d). Reciprocally, VCP prevents excessive calcium entry in mitochondria by regulating the mitochondrial calcium intake through the degradation of mitochondrial calcium uptake (MICU) proteins (e), which subsequently inhibits mPTP opening, preventing cell death. Impairment of VCP activity induces ER and mitochondrial dysfunctions, which subsequently induce cellular damage, resulting in the pathogenesis of the diseases.

In summary, as shown in Figure 2, VCP plays an essential role in maintaining cellular ER and mitochondrial function and calcium homeostasis in normal cells, and thus, impairment of the VCP activity, caused by deficiency of expression or the mutation of structure, will induce ER and mitochondrial dysfunctions, which subsequently induce cellular damage, resulting in the pathogenesis of the diseases. 

## 4. The Aberrant Expression of VCP Mediates ER and Mitochondria Dysfunctions in the Progress of Various Cancers 

Accumulating evidence from clinical studies has revealed the prognostic value of VCP in a wide range of cancers (Table 1), and demonstrated an enhanced ERAD and upregulated VCP expression in cancer cells [42]. For example, VCP upregulation has been associated with unfavorable clinical outcomes in breast cancer patients, and was found to be involved in cell growth and survival in colorectal cancer [43]. In other malignancies in the lung, prostate and pancreas, the VCP expression level was correlated with recurrence rate and prognosis in the patients [44]. Interestingly, although the aberrant expression of VCP has been shown to contribute to cancer progression at different stages, the role of VCP in the pathogenesis of cancers appears controversial. For instance, VCP expression declines in osteosarcoma cell lines, however, VCP expression is continuously increased in its subline LM8 cells, which display higher metastatic potential [45]. Yamamoto et al. reported that in gastric carcinoma and in esophageal squamous cell carcinoma (ESCC), a higher expression of VCP indicates a higher rate of lymph node metastasis, deep tumor invasion, and poorer overall and disease-free survival [46,47]. However, in human papilloma virus (HPV)-negative oropharyngeal squamous cell carcinoma (OSCC), high expression of VCP was related to significantly better five-year disease-free survival. It has also been reported that in HPV-negative OSCC, patients who lose the chromosome region 9p13-p12, in which the VCP gene is located, display a poor survival rate [48]. 

VCP expression is upregulated in most types of cancer, and the level of VCP expression is genitively associated with survival rates in patients. However, the correlation varies in some types of cancers.

Several studies have revealed the potential mechanisms of VCP involved in cancer cell reprogramming. The VCP-interacting motif is regulated mainly by three proteins: E3 ubiquitin-protein ligase gp78, the recruitment factor for E3 ubiquitin-protein ligase sialoprotein S (VIMP), and the small VCP/p97-interacting protein (SVIP) [52,53]. While Gp78 and VIMP have been shown to promote ERAD in cancer cells, SVIP has been reported to play an opposing role, and exert an inhibitory effect on this process [54]. In many human tumors, SVIP is silenced [42], resulting in an upregulation of VCP, which in turn facilitates ERAD to avoid programmed cell death. These studies have provided the potential mechanism by which cancer cells avoid ER stress-induced programmed cell death, and become progressive in a VCP-dependent manner.

Evidence also indicates that VCP plays an important role in the metastatic process in cancer cells, by involvement in the ubiquitin-dependent proteasome degradation pathway via the Akt/NFκB pathway [55]. In addition, recently, ER stress has been reported to induce epithelial–mesenchymal transition (EMT) in cancers, which is critical for the progression and metastasis of the tumor. During the process of EMT, epithelial markers are decreased, while the mesenchymal markers are increased in the cells. ER stress level is the driving force of cell fate—either apoptosis or EMT [56]—and also induces abnormal cell differentiation and morphological changes [57]. Findings from the recent study suggest that, at the early stage of cell dysfunction, loss of VCP results in elevated ER stress, which may transform cells into an EMT-like state. However, prolonged deficiency of VCP will lead to programmed cell death [58].

Mitochondria have been also shown to be critical in cancer transformation, progression and metabolism [59]. The dysfunction of mitochondria has been related to many pathological processes in human cancers [60]. Mitochondrial quality control dysregulation causes mitochondria stress, and increases reactive oxygen species (ROS) production, which may be involved in cancer predisposition [61]. In the mitochondria, the quality control of the outer membrane (OMM) proteins plays an important role in the maintaining of mitochondria homeostasis. Many OMM proteins are regulated by the ubiquitin–proteasome system, in which VCP plays a pivotal role [29,62]. For example, VCP/Cdc48-associated mitochondrial stress-responsive 1(Vms1), a mitochondrial protein, translocates from the cytoplasm to mitochondria during mitochondria stress, and plays an important role in regulating mitochondria quality control in a complex with VCP, through the ubiquitin–proteasome system. The decrease of Vms1 impairs the degradation of the OMM protein and induces mitochondria stress [63]. Thus, as a major regulator of mitochondria, VCP could also play critical roles in the pathogenesis of cancers.

Based on these results, VCP is now considering as an important target for cancer treatment. It has been shown that the inhibition of VCP is able to induce proteotoxic stress and apoptosis in cancer cells, and improve radiation sensitivity in ESCC [64]. One of the VCP inhibitors, CB-5083, is the first selective VCP inhibitor to show preclinical effects [13]. However, the clinical trial of CB-5083 failed due to its toxicities. Some new compounds that may have better potential effects are being investigated, like the VCP inhibitor compound 35, in non-small cell lung cancer [44].

## 5. VCP Structural Mutations Induce ER and Mitochondrial Impairments Resulting in Neuro-Degenerative Disorders

The mutations of VCP have been associated with diverse myodegenerative and neurodegenerative disorders including inclusion body myopathy (IBM), with Paget disease of the bone, as well as frontotemporal dementia (IBMPFD) and amyotrophic lateral sclerosis (ALS).

IBMPFD is a multisystem degenerative disorder caused by mutations in VCP, which includes IBM associated with Paget’s disease of the bone (PDB) and frontotemporal dementia (FTD) [65]. IBM is a hereditary disease with uncertain pathogenic mechanisms, and it is usually associated with progressive asymmetric muscle weakness and other pathological features, including invasion of myofibers by cytotoxic T cells, congophilic inclusions, cytoplasmic aggregates and rimmed vacuoles [66]. One of the characteristic hallmarks of this disorder is cytoplasmic protein aggregation, which is associated with a large range of proteins, such as ubiquitin, amyloid-β peptides, phosphorylated tau, RNA-binding protein, TAR-DNA binding protein 43 (TDP-43) and prion protein, which results in imbalanced protein homeostasis in those diseases [67,68]. Multiple mutations of VCP have been demonstrated to contribute to the pathogenesis of IBMPFD. For instance, multiple sites of mutations at R95G, R155P, R155C and R155H have been observed in the N-domain of VCP, while R155H is the most frequently identified mutation. Studies have shown that expressing VCP R155H in cultures cells results in an abundant accumulation of misfolded substrates, suggesting ERAD is severely disrupted in IBMPFD [69]. Other mutations were confirmed to be located in the N- to D1-domain linker and D1 domain, such as R191Q and A232E [65], which altered the structural orientation in N and/or D1 domains, consequently affecting its cofactor-binding features and ATPase activity [70,71].

Studies have postulated the potential molecular mechanisms by which VCP regulates IBMPFD pathogenesis. First, the IBMPFD-associated mutations in VCP disrupt the ubiquitin-conjugated protein turnover in the ER membrane and aggregate with other cofactors, ultimately impairing the UPS pathway. These phenomena may be attributed to the interruption of the protein–protein interactions in the ERAD pathway, which impairs the transferring of ubiquitinated proteins from ER to proteasome [69]. Second, overexpressing VCP mutants, VCP-R155H or VCP-A232E, results in defects in autophagy by impairing ubiquitin-containing autophagosome maturation, and leads to the accumulation of autophagosomes, which contribute to the pathogenesis of IBMPFD [65]. Third, VCP acts as an important part in the Shoc2– Ras–RAF-1 complex, in regulating the ERK1/2 signaling pathway [72]. VCP mutations disturb ERK1/2 phosphorylation and alter ERK1/2 activity, which may play an important role in IBMPFD pathogenesis. Finally, VCP, interacting with the endoplasmic reticulum (ER) morphology regulator Atlastin-1(ATL1), regulates dendritic spine formation in neurons by influencing tubular ER formation and protein synthesis [24].

ALS, a rapid progress disease caused by degeneration of motor neurons (MNs), is another hereditary disease associated with VCP mutations. Mislocalization and aggregation of TDP-43 in the cytoplasm has been observed in VCP-mutated MNs, accompanied with an increased Bip and p-eIF2alpha and a decreased ER calcium storage [73]. TDP-43 dislocation and aggregation have also been connected with mitochondrial quality control defects, which may disturb the ER–mitochondria contacts and prevent protein import to the mitochondria matrix [74]. These studies have suggested that aberrant VCP expression may be associated with the pathogenesis of this disorder.

Mitochondrial function is also found to be disturbed by VCP mutations in neurodegeneration diseases like ALS and IBMPFD, including the increased mitochondrial fragmentation, the loss of mitochondrial membrane potential and the increased reactive oxygen species (ROS). VCP, as the ubiquitin-dependent chaperone, maintains the mitochondrial function in neuronal cells through the UPS and the outer mitochondrial membrane-associated degradation (OMMAD) [75]. In ALS, VCP mutations disrupt the damaged mitochondria clearance through impairing the PINK1/Parkin pathway [62].

## 6. VCP Represents a New Regulator in Cardiac ER and Mitochondria Functions, and is Involved in Various Heart Diseases

Of note, VCP was identified to be expressed in the heart [18]. Moreover, studies in Drosophila have also revealed that the knockdown of TER94 (VCP homolog in Drosophila) with cardiac-restricted siRNA severely disrupts myofibrillar organization and heart function in adult flies [76]. Taken together, these findings highlight the importance of VCP in the heart.

Clinical observation found that dilated cardiomyopathy commonly existed in patients with IBMPFD [77]. Cardiac-specific overexpression of an enzymatically mutated VCP (VCPK524A) in transgenic mice resulted in an impairment of the ATPase activity within the D2 domain of VCP, and lead to the development of cardiomyopathy [78]. These mice displayed an ERAD dysfunction and an accumulation of ubiquitinated proteins in the heart.

In addition, recent studies have revealed that the reduction of VCP expression was associated with hypertensive heart diseases [16]. For example, VCP expression was found to be significantly decreased in the heart of spontaneously hypertensive rats (SHR), compared to their normotensive Wistar Kyoto (WKY) controls. VCP expression was also found to be progressively decreased in the mouse hearts, along with persistent pressure overload [16]. Reciprocally, cardiac-specific overexpression of VCP attenuated the pressure overload-induced cardiac pathogenesis [19]. These phenomena are linked to the inhibitory effects of VCP on the transduction of the AKT/mTOC1R/S6K signaling pathway [16]. It is also found that the disruption of VCP ATPase activity results in age-dependent cardiomyopathy, because of the defect in the ubiquitinated targeted proteins’ degradation, mediated by UPS [78]. This suggests the critical role of VCP in regulating ER function in aging- and pressure overload-related cardiac cardiomyopathy.

Furthermore, increasing evidence indicates that VCP plays an important role in regulating mitochondrial function in the heart. The heart is an organ with high energy demands, and approximately 95% of energy consumed by the heart is from mitochondria [79]. Mitochondrial dysfunction has been observed in a variety of heart diseases, particularly in conditions with reduced blood supply and oxygen, such as ischemic heart injury [80]. Recent studies have demonstrated that cardiac-specific overexpression of VCP can protect the heart against ischemia/reperfusion injury, and prevent cell death in an inducible nitric oxide (NO) synthase (iNOS)-dependent manner [17]. These protective effects of VCP are related to the preservation of the cardiac mitochondrial function and the inhibition of the opening of mitochondrial permeability transition pores, through inhibiting the stress-induced excessive calcium uptake by repressing MICU1, and activating MICU2, which may be associated with the selective degradation of the mitochondrial protein by VCP [81]. Moreover, VCP is also identified as a link between NF-κB-induced iNOS expression and the AKT signaling pathway, which is activated by the heat shock protein22 (HSP22) [18], which is also a key regulator of UPS [82] and mitochondrial function [83]. However, despite these significant findings, the role of VCP in the pathogenesis of cardiomyopathy, and the mechanisms involved, remain largely unknown.

## 7. Future Directions

**Figure 3:**
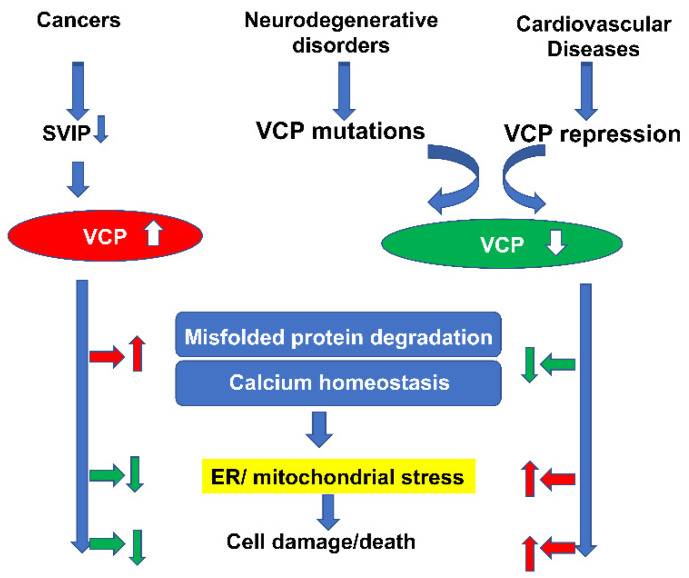
Summary of the common features related to VCP in different diseases. Despite the fact that the cofactors and the regulatory signaling pathways underlying the pathogenesis of these disorders may vary, VCP could promote stress-induced misfolded protein degradation and maintain calcium homeostasis, which subsequently helps resist ER/mitochondrial stress-induced cell damage and death. As the increase of VCP in cancer cells would protect cells against death, the deficiency of VCP in neurodegenerative disorders and heart diseases would induce cell damage and death, due to the loss of these functions. (The red arrows represent the promotive effects and green arrows stand for the inhibitory/suppressive effects).

In this review, we have summarized the involvement of VCP in a variety of pathological conditions, particularly in cancers, neurodegenerative disorders and heart diseases. Although the regulatory signaling pathways underlying the pathogenesis of these disorders are not fully understood, and may vary among the different diseases, there are some common features related to VCP in these disorders. For example, as shown in Figure 3, by interacting with different cofactors/adaptors, VCP could promote stress-induced misfolded protein degradation and maintain calcium homeostasis, which subsequently helps resist ER/mitochondrial stress-induced cell damage and death. Thus, the increase of VCP in cancer cells would protect cells against death, while the deficiency of VCP in neurodegenerative disorders and heart diseases would induce cell damage and death, due to the loss of these functions. This evidence has highlighted the role of VCP in mitochondrial and/or ER-related disorders.

Therefore, future research could draw more attention to the definition of the detailed mechanism of VCP that is involved in the regulation of ER and mitochondrial interaction, in calcium metabolism and homeostasis. In addition, since VCP’s effects largely rely on its banded cofactors, exploring the potential interactions between VCP and other proteins, particularly the cofactors and substrates of the N-terminal domain of VCP, will bring new insight into the molecular mechanism in VCP-related diseases. Furthermore, VCP-mediated protein subcellular translocation is crucial in ER and mitochondrial function, and further understanding the mechanism by which VCP mediates other protein translocations will help to define the new effect of VCP in integrating homeostasis among multiple organelles. It is also necessary to explore the mechanisms that stimulate VCP in cancer cells, or their reduction under cardiac stress, which will lead to the discovery of the pathogenesis of the related diseases. Finally, it is important to design and develop VCP inhibitors for the treatment of various cancers, and activators of VCP to restore the VCP in stressed hearts, to prevent cardiomyopathy.

## Figures and Tables

**Table 1:** Summary of VCP expression and prognostic value in different types of cancer.

Cancer Type	Factor	No. of Patients	Survival Rate	*p* Value
Follicular Thyroid Cancer [14]	VCP expression		10-y disease-free of survival	<0.01
	Low	33	96.80%	
	Intermediate	19	66.20%	
	High	20	80.00%	
Gingival squamous cell carcinoma [49]	VCP expression		5-y disease-free of survival	<0.05
	Low	24	86.3	
	High	50	63.9	
Breast carcinoma [50]	VCP expression		5-year overall survival rate	<0.001
	Negative	49.50%	higher	
	Positive	50.60%	lower	
HPV-negative OSCC [48]	VCP expression		5-year disease-free survival	0.017
	Low	29	45.60%	
	High	37	86.40%	
hepatocellular carcinoma [51]	VCP expression		5-year disease-free	<0.01
	Low	57	higher	
	High	105	lower	
gastric carcinoma [46]	VCP expression		5-year disease-free	<0.001
	Low	94	higher	
	High	233	lower	
Esophageal Carcinoma [47]	VCP expression		5-year disease-free survival rates	<0.001
	Low	57	65.90%	
	High	96	33.20%	
non-small-cell lung carcinoma [15]	VCP expression		5-year disease-free	<0.05
	Low	69	higher	
	High	135	lower	
